# Functional Connectivity of the Corpus Callosum in Epilepsy Patients with Secondarily Generalized Seizures

**DOI:** 10.3389/fneur.2017.00446

**Published:** 2017-08-30

**Authors:** Syu-Jyun Peng, Yue-Loong Hsin

**Affiliations:** ^1^Institute of Electronics, National Chiao Tung University, Hsinchu, Taiwan; ^2^Biomedical Electronics Translational Research Center, National Chiao Tung University, Hsinchu, Taiwan; ^3^Department of Neurology, Chung Shan Medical University and Chung Shan Medical University Hospital, Taichung, Taiwan

**Keywords:** corpus callosum, resting-state function MRI, functional networks, regional homogeneity, secondarily generalized seizures

## Abstract

The corpus callosum (CC) plays an important role in generalization of seizure activity. We used resting-state function magnetic resonance imaging (rs-fMRI) to investigate the regional and interregional functional connectivity of CC in patients with magnetic resonance imaging (MRI)-negative and secondarily generalized seizures. We measured the multi-regional coherences of blood oxygen level-dependent (BOLD) signals *via* rs-fMRI, cortical thickness *via* high-resolution T1-weighted MRI, and white matter (WM) integrity *via* diffusion-tensor imaging in 16 epilepsy patients as well as in 16 age- and gender-matched healthy subjects. All patients had non-lesional MRI, medically well-controlled focal epilepsy and history of secondarily generalized convulsions. Individuals with epilepsy had significant differences in regional and interregional hypersynchronization of BOLD signals intrahemispherically and interhemispherically, but no difference in cortical thickness and WM integrity. The only area with increased regional hypersynchrony in WM was over the anterior CC, which also exhibited lower activation of neighboring resting-state networks. The present study revealed abnormal local and distant synchronization of spontaneous neural activities in epileptic patients with secondarily generalized seizures.

## Introduction

When a seizure evolves into a generalized convulsion, the electro-clinical features, including bilateral stiffening, jerking of limbs, and synchronization of electroencephalography (EEG) discharges, are not very different from those of most generalized seizures in and among epilepsy patients. Sequential involvement of different cortices and their associated neural fibers for the widespread propagation of epileptic activity is a generally accepted scenario for the development of high amplitude, bilaterally synchronous EEG discharge from a period of locally repetitive spiking (1).

The corpus callosum (CC) is a commissure fiber bundle in the brain that connects bilateral cerebral hemispheres. The widespread cortical connection through the CC is assumed to implicate the interhemispheric transmission of epileptic electricity. Therefore, callosotomy, a surgical procedure, may be conducted to disconnect the bilateral hemispheres to reduce the severity and frequency of secondary generalization in patients with pharmacoresistant focal seizures (2). During the generalized seizure period, the limited temporal difference between the EEG spiking ridges of remote cerebral regions indicates that there is an effective process to synchronize the interhemispheric electrical activity independent of synaptic transmission, gap junctions, and ion diffusion. In addition to the synchrony disruption, callosotomy also decreases the intrahemispheric temporal coupling between cortices, supporting the facilitation mechanism of the CC (3).

Modern techniques of magnetic resonance imaging (MRI) acquisition and processing enable researchers to investigate the structural and functional networks of patients with different epileptic syndromes to identify the mechanisms and progression of seizures as well as their comorbidities. Diffusion-tensor imaging (DTI) allows computation of microstructural properties of the nerve fibers. Changes of DTI parameters, such as the fractional anisotropy (FA) value, have been found diffusely in the brains of patients with idiopathic-generalized epilepsies (IGEs) and mesial temporal lobe epilepsy (mTLE) through group comparisons mostly using voxel-wise statistical analyses (4, 5). As mentioned, research addressing abnormal networks in the epilepsies is also needed to measure functional interactions or synchronization between different brain regions for ictogenesis and seizure propagation. Here, resting-state functional magnetic resonance imaging (rs-fMRI) is a suitable tool for investigating functional networks at the whole-brain macroscale level, as it is based on the temporal correlations of low-frequency fluctuations (<0.1 Hz) of blood oxygen level-dependent signals (BOLD) (6). The common methods used in this paradigm include the region of interest (ROI)-based approach, the independent component analysis (ICA) to assemble the distant functional networks, and the regional homogeneity (ReHo) assessment to characterize the local connectivity. Using these methods, altered different functional networks have been found in patients with IGEs and mTLE (7, 8).

Nevertheless, these network studies have rarely examined the association between local and remote connections or modeled the structure–functional relationships in the CC. The purpose of this study was to investigate the association between secondarily generalized seizures and altered function or structural brain connectivity. From a medically well-controlled patient group with a history of generalized convulsions, a localizable seizure source and without visible lesions on MRI, we studied structural connectivity by measuring the white matter (WM) integrity *via* DTIs and the functional connectivity *via* rs-fMRI. First, we employed tract-based spatial statistics (TBSS) to analyze the differences of WM FA values between patients and normal controls. Then, we applied the ROI-based, ICA, and ReHo methods to the rs-fMRI data to resemble functionally relevant cortical network modes and define certain locations with highest regional synchronization of activity. In addition, we compared the cortical thickness of patients with controls through T1-weighted MRI to estimate the dependence of functional connectivity change on cortical differences. We attempted to investigate any alterations of functional and structural networks that would be restrained by the CC.

## Materials and Methods

### Subjects

We retrospectively reviewed the clinical database of Hualien Tzu Chi General Hospital to collect MR data of patients with secondary generalization of focal seizures. We excluded patients considered “lesional” when the radiologists had identified any questionable lesions on MRIs (for examples, possible sclerotic or atrophic changes in mesial temporal lobe; or with possible blurred gray–white matter junction). Patients who possibly had mTLE with maximal interictal or ictal epileptiform discharges localized to the temporal lobe and with classically semiological features of mTLE were excluded. Patients who probably had conventionally termed primarily generalized epilepsies with symmetric interictal or ictal epileptiform discharges and with non-lateralized presentations of clinical seizures were also excluded. Seizure videos or medical records were required to support focal onset with evolution of general convulsion. Recruited patients were categorized as having medically well-controlled epilepsy only with rare seizure recurrence (less than once every 6 months) under antiepileptic monotherapy. MRI data from 16 patients (6 males and 10 females, age = 32.7 ± 10.5 years) fitting our inclusion criteria were collected. One patient had occipital lobe epilepsy and exhibited elementary visual hallucination at seizure onset. Two patients had parietal lobe epilepsy with paresthetic sensation at onset. Six patients had a seizure focus over the left frontotemporal regions and exhibited a cessation of speech at onset or postictal aphasia. Seven patients had maximal seizure EEG activity over the right or bilateral frontopolar regions at onset (Table 1). Sixteen healthy controls without any history of neurological or psychiatric symptoms that were matched in age and gender (6 males and 10 females, mean age = 33.1 ± 10.1 years) to the seizure patient group were recruited. The study protocol, which consisted of retrospectively analyzing the images of the patients and collecting images from the control subjects, was approved by the ethics committee of Buddhist Tzu Chi General Hospital in Hualien, Taiwan, and written informed consent was obtained from each participant.

**Table 1:** The demographic and clinical data.

Case	Gender/age	Age of onset	Seizure semiology	Electroencephalography	Pharmacotherapy
1	M/22	19	CPS	Rt F	CBZ
2	F/41	22	CPS	Lt F	VPA
3	F/26	20	CPS	Rt F	TPM
4	F/35	19	CPS	B F/T	PHT
5	F/41	25	CPS	Lt F	LVT
6	M/21	17	CPS	Rt O	OXC
7	F/23	17	CPS	Rt F	LMT
8	F/46	19	CPS	Lt F/T	OXC
9	F/33	21	SP	Rt F/T	LMT
10	F/57	22	CPS	Lt F	VPA
11	M/30	24	CPS	Rt F	VPA
12	F/44	18	CPS	Rt F	LMT
13	F/26	18	CPS	Lt F	LMT
14	M/22	19	SP	Rt P	OXC
15	M/27	12	CPS	Lt F/T	OXC
16	M/29	20	SP	Lt P	OXC

*Lt, left; Rt, right; B, bilateral; F, frontal; T, temporal; P, parietal; O, occipital; CPS, complex partial seizure; SP, simple partial seizure; CBZ, carbamazepine; LMT, lamotrigine; OXC, oxicarbazepine; PHT, phenytoin; TPM, topiramate; VPA, valproic acid; LVT, levetiracetam*.

### Image Acquisition

Since 2010, patients with epilepsy have been studied with an epilepsy-specific imaging protocol that includes an acquisition section for rs-fMRI and standard structure imaging sections. For this study, the control subjects were imaged with the same imaging sequences. MRI was performed on a SIGNA HDX 1.5T (GE, Milwaukee, WI, USA). A high-resolution T1-weighted image was acquired using a fast-spoiled gradient-recalled echo sequence: repetition time (TR) = 14.024 ms; echo time (TE) = 14.024 ms; flip angle = 15°; field of view (FOV) = 220 mm × 220 mm; matrix = 256 × 256; and slice thickness = 1 mm. The DTIs were acquired with the following parameters: TR = 8,000 ms; TE = 82.4 ms; flip angle = 15°; FOV = 250 mm × 250 mm; matrix = 256 × 256; slice thickness = 3 mm; number of excitations = 2; 25 gradient directions with a *b* value of 1,000 s/mm^2^; and 1 null tensor image with a *b* value of 0 s/mm^2^. The rs-fMRI data were obtained by: TR = 2.223 ms; TE = 35 ms; flip angle = 90°; FOV = 240 mm × 240 mm; matrix = 64 × 64; slice thickness = 4 mm; and totaling 6 min 40 s per scan.

### Analyses of Structural MRI

We computed a FA map using the FMRIB Software Library 4.1.9 package^1^ (9). Tract-Based Spatial Statistics (TBSS) of FA maps was performed using the TBSS tool in the FMRIB Software Library (10). Then, voxel-wise group comparisons were carried out using non-parametric, two-sample *t*-tests between the patient and control groups after controlling for the effects of age and gender. The mean FA skeleton was used as a mask. The significance threshold for between-group differences was set at *p* < 0.05 (Familywise Error Rate corrected for multiple comparisons) using the threshold-free cluster enhancement option in the “randomize” permutation-testing tool in FSL.

Cortical thickness was calculated using the FreeSurfer software package^2^ (11). FreeSurfer was used to spatially register the cortical thickness maps into the FreeSurfer standard space. These maps were then used to perform general linear model (GLM) analysis for group comparisons and to determine the predictors for cortical thickness variations. Thickness maps were smoothed with a 10-mm full-width at half-maximum (FWHM) Gaussian kernel. The age, gender, and total intracranial volume were used as covariates. Using the built-in GLM tool in FreeSurfer, Qdec, we compared the cortical thickness between groups. All Qdec results were corrected for multiple comparisons used in the assessment of the cluster size *p*-values. These results, which were corrected for multiple comparisons, were considered significant at *p* < 0.05.

### Analyses of Functional MRI

The rs-fMRI data for each subject was preprocessed by the following steps. The first 10 scans of rs-fMRI data were removed to allow magnetization equilibrium. The slice-timing correction was based on the middle slice. The rs-fMRI data were realigned to the first dynamic scan by rigid body correction. Six parameters of the head movements were generated. Participants who exhibited head motion >1 mm of translation or 1° of rotation were excluded. There are no differences in movement between patients and controls. Finally, the data were then normalized to the MNI space with voxel size resampling of 3 × 3 × 3 using the DARTEL toolbox (12).

For the ReHo analysis, the preprocessed rs-fMRI data were band-pass filtered within 0.01–0.08 Hz following a linear detrend to reduce the effects of high-frequency noise and low-frequency drift. ReHo analysis was performed for each subject by calculating the Kendall’s coefficient of concordance of the time series of a given voxel with those of its nearest neighbors (26 voxels) in a voxel-wise analysis (13). The intracranial voxels were extracted to make a mask. For standardization purposes, each individual ReHo map was divided by its own mean ReHo within the mask and then spatially smoothed with a Gaussian kernel of 4 mm FWHM. To identify the differences in the ReHo between patient and control groups, we performed a *t*-test comparison between the two groups, and a double statistical threshold was used (combined height threshold *p* < 0.05 and a minimum cluster size = 54 voxels, as determined by the AlphaSim correction by REST software) (14).

For the ROI-based network analyses, the preprocessed rs-fMRI data were spatially smoothed with a Gaussian kernel of 4 mm FWHM and band-pass filtered within 0.01–0.08 Hz following a linear detrend. To remove spurious signals that were unlikely to reflect neural activity, we controlled for several nuisance covariates in the linear regression, including the six head motion parameters, global mean signal, WM signal, and cerebrospinal fluid signal. We obtained the mean time series of each of the 116 regions of the Anatomical Automatic Labeling template by averaging the fMRI time series over all voxels in the region (15, 16). Correlation coefficients were computed between each pair of all 116 regions. Then, a Fisher’s *r*-to-*z* transformation was applied to improve the normality of these correlation coefficients. The individual *z* values were entered into a random effect one-sample two-tailed *t*-test to determine brain regions showing significant correlations within each group. These *z* values were also entered into a random effect two-sample two-tailed *t*-test to determine the regions showing differences in correlations between the groups. The functional correlations that were considered significantly different between the patient and control group must satisfy two criteria: (1) there were significantly different *z* values between the two groups at the threshold of *p* < 0.01 (uncorrected) and (2) the *z* values of the correlations were significantly different from 0 in at least one group at a threshold of *p* < 0.01 (uncorrected).

For the ICA analysis, the preprocessed rs-fMRI data were spatially smoothed with a Gaussian kernel of 4 mm FWHM. Group spatial ICA was used to decompose all the data into independent components using the GIFT software^3^ (17). The time-courses and spatial maps of the independent components for each participant were acquired for subsequent processing. The subject-specific maps created from a corresponding group-level IC by back-projection were converted to *z*-scores. Both the spatial pattern and frequency spectra of each component were visually inspected to determine their appearance as potential resting-state networks (RSNs) or possible image artifacts. Three RSNs, the executive control network (ECN), sensorimotor network (SMN), and default mode network (DMN), which spatially associated with prefrontal and premotor cortexes, were of interest and were thus selected for further analyses.

For the RSNs, *z*-maps in each group were then gathered for a random-effect analysis using the one-sample *t*-test (combined height threshold *p* < 0.001 and a minimum cluster size = 100 voxels, as determined by the AlphaSim correction by REST software). Subsequently, to investigate the functional connectivity changes in the ECN, SMN, and DMN, the *z*-maps of the RSNs were compared between groups using two-sample *t*-tests (combined height threshold *p* < 0.001 and a minimum cluster size = 6 voxels, as determined by the AlphaSim correction by REST software) (14). Particularly, in each RSNs, we restricted the two-sample *t*-test to include only the voxels within a mask that was defined by the one-sample *t*-test of RSN of the subjects in the control group.

## Results

### Group Differences in Structural Networks

TBSS did not reveal any differences on the diffusion map skeletons of FA between the patients and controls. There was no difference in the cortical thickness between the patient and control groups.

### Group Differences in Regional Synchronization

**Figure 1:**
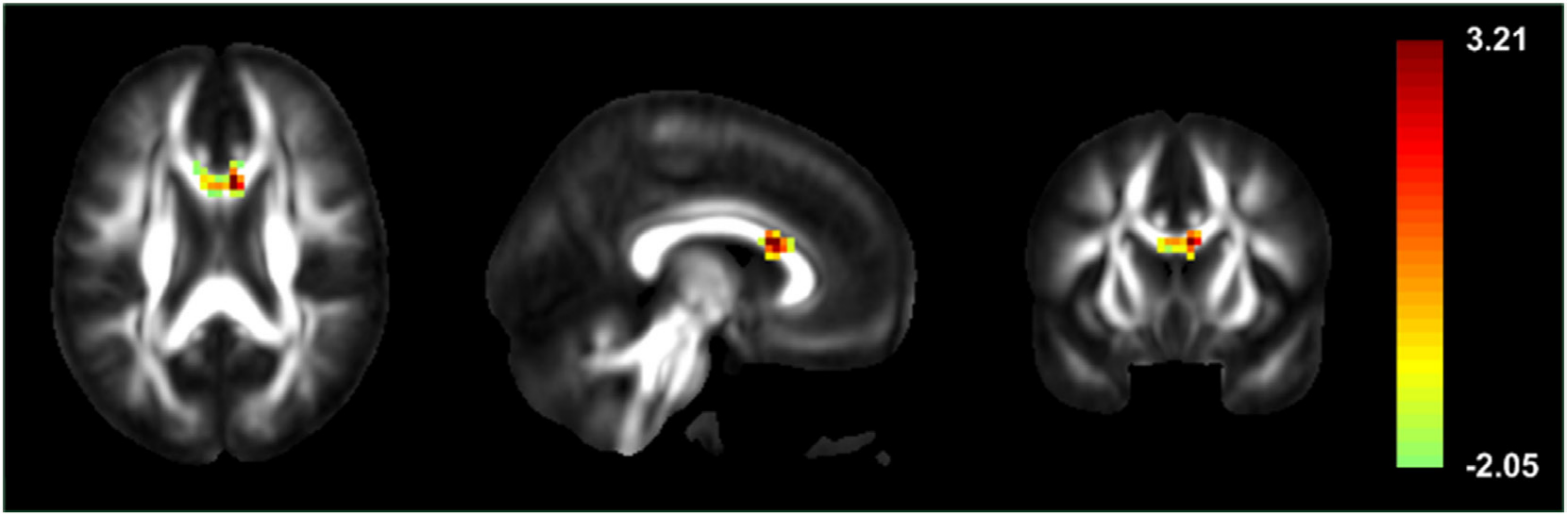
Statistic *t*-map illustrating high local synchronization of blood oxygen level-dependent signals in the genu of the corpus callosum of patients with secondarily generalized seizures, when compared to healthy controls.

Patients showed significantly increased ReHo compared to healthy controls in the genu of CC, right superior temporal pole, right lingual gyrus, left lobule VI of the cerebellar hemisphere, lobule III of the vermis, and left putamen. Significant ReHo decreases were seen in the right inferior temporal gyrus, right midcingulate area, left inferior occipital cortex, left medial frontal gyrus, and left postcentral gyrus (Figure 1; Table 2).

**Table 2:** Regions with significant regional homogeneity differences.

Regions	MNI coordinate	Peak *t*-score	Number of voxels
Genu of corpus callosum	−6, 12, 21	3.21	54
R superior temporal pole	45, 9, −18	3.65	84
R lingual gyrus	3, −78, −9	4.43	527
Left lobule VI of cerebellar hemisphere	−27, −45, −21	3.21	66
Lobule III of vermis	3, −42, −9	3.37	64
L putamen	−21, 12, 9	4.39	64
R inferior temporal gyrus	63, −57, −9	−3.73	74
R precentral gyrus	51, 0, 30	−4.17	208
R midcingulate area	6, −30, 39	−4.16	104
L inferior occipital cortex	−24, −90, −3	−3.73	89
L medial frontal gyrus	−12, 60, 18	−4.14	113
L postcentral gyrus	−63, −15, 24	−3.42	60

*The values given are the stereotactic MNI coordinates and the *t*-score of each anatomical region. SPM maps were thresholded at *p* < 0.05 (AlphaSim correction, voxel-level, minimum cluster size threshold of 54 voxels)*.

*R, right; L, left; MNI, Montreal Neurological Institute*.

### Group Differences in Interregional Correlations

**Figure 2:**
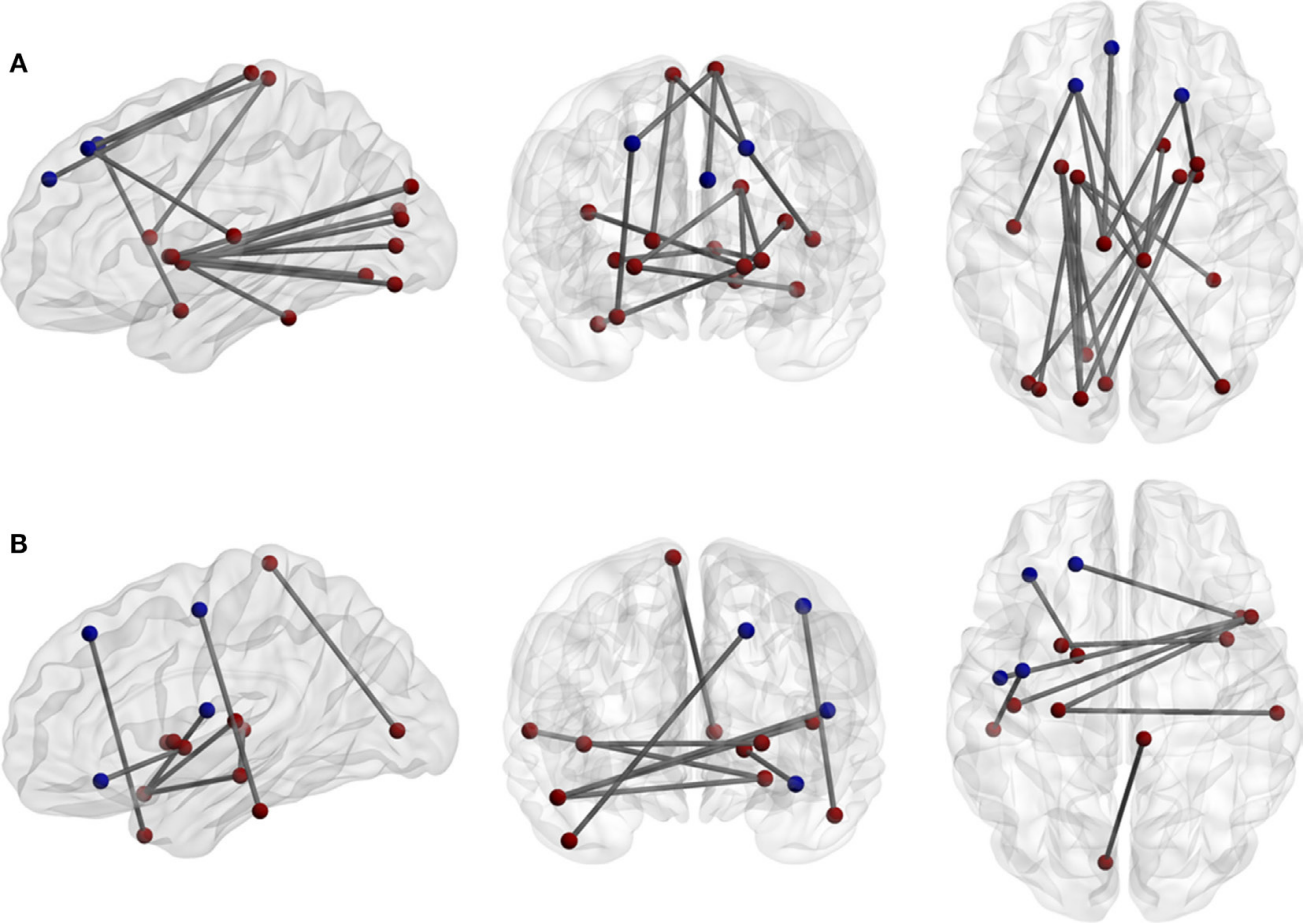
**(A)** Increases and **(B)** decreases of interregional correlations in the patient group. The network density of **(A)** is higher than **(B)**. The blue circuits represent anatomical regions of interest (ROI) from the automated anatomical labeling atlas across the prefrontal and premotor regions.

In the ROI-based network analysis, the whole brain was divided into 116 regions, and we identified abnormal connectivity by comparing the correlation coefficients of each pair. There were 24 significantly increased and 13 significantly decreased interregional correlations between the patients and the healthy controls. Excluding the interrelationship with the cerebellum, 7 of 18 increased correlations and 7 of 9 decreased correlations were interhemispheric. Three of six prefrontal regions correlated with contralateral cortices (Figure 2; Table 3). However, there were no significant differences of correlation coefficients of each ROI pair for multiple comparisons between the patients and the healthy controls.

**Table 3:** The interregional correlations.

Increased interregional correlation				Decreased interregional correlation			
Classification	Region 1	Classification	Region 2	Classification	Region 1	Classification	Region 2
Prefrontal	L.SFGdor	Parietal	L.PCL	Prefrontal^a^	L.SFGdor	Temporal	R.TPOmid
Prefrontal^a^	L.SFGdor	Parietal	R.PCL	Prefrontal	L.ORBinf	Corpus striatum	L.PAL
Prefrontal	L.SFGdor	Temporal	L.HES	Prefrontal^a^	R.ACG	Cerebellum	R.CER3
Prefrontal^a^	L.SFGdor	Cerebellum	R.CER3				
Prefrontal	R.SFGdor	Temporal	R.AMYG	Other parts of frontal lobe^a^	L.ROL	Temporal	R.TPOsup
Prefrontal^a^	R.SFGdor	Parietal	L.PCL	Other parts of frontal lobe	L.PreCG	Temporal	L.ITG
Prefrontal^a^	L.IFGtriang	Cerebellum	R.CER9	Other parts of frontal lobe^a^	R.DCG	Cerebellum	L.CER9
Prefrontal	L.SFGmed	Parietal	L.PCL				
Temporal	L.FFG	Cerebellum	L.CER3	Temporal^a^	L.HIP	Temporal	R.STG
Temporal^a^	R.FFG	Corpus striatum	L.PAL	Temporal^a^	L.HIP	Temporal	R.TPOsup
Temporal^a^	R.FFG	Cerebellum	L.CER9	Temporal^a^	L.HES	Temporal	R.TPOsup
Temporal^a^	R.ITG	Cerebellum	L.CER3	Temporal	R.HES	Cerebellum	R.CER3
Parietal	R.PCL	Corpus striatum	R.CAU	Temporal	R.HES	Cerebellum	R.CER4 and 5
Parietal	R.PCL	Cerebellum	R.CER8	Parietal	L.PCL	Cerebellum	L.CER4 and 5
Occipital	L.CAL	Corpus striatum	L.PUT	Parietal^a^	L.PCL	Cerebellum	R.CER4 and 5
Occipital^a^	L.CAL	Corpus striatum	R.PUT	Occipital^a^	L.CAL	Parietal	R.PCL
Occipital	L.LING	Corpus striatum	L.PUT				
Occipital^a^	L.LING	Corpus striatum	R.PAL	Corpus striatum^a^	L.CAU	Cerebellum	R.CER10
Occipital	L.SOG	Corpus striatum	L.PUT	Cerebellum	R.CER4 and 5	Cerebellum	R.CER10
Occipital	L.SOG	Corpus striatum	L.PAL	Cerebellum	L.CER10	Vermis	VER4 and 5
Occipital^a^	L.SOG	Corpus striatum	R.PAL				
Occipital	L.MOG	Corpus striatum	L.PAL	Insula^a^	R.INS	Corpus striatum	L.PUT
Occipital^a^	R.MOG	Corpus striatum	L.PAL	Insula	R.INS	Cerebellum	R.CER3
Occipital^a^	L.IOG	Corpus striatum	R.PAL				

*^a^Interhemisphere*.

*L, left; R, right; ACG, anterior cingulate and paracingulate gyri; AMYG, amygdala; CAL, calcarine fissure and surrounding cortex; CAU, caudate nucleus; CER3, cerebellum 3; CER4 and 5, cerebellum 4 5; CER8, cerebellum 8; CER9, cerebellum 9; CER10, cerebellum 10; DCG, median cingulate and paracingulate gyri; FFG, fusiform gyrus; HES, Heschl gyrus; HIP, hippocampus; IFGtriang, inferior frontal gyrus, triangular part; INS, insula; IOG, inferior occipital gyrus; ITG, inferior temporal gyrus; LING, lingual gyrus; MOG, middle occipital gyrus; ORBinf, inferior frontal gyrus, orbital part; PAL, pallidum; PCL, paracentral lobule; PreCG, precentral gyrus; PUT, lenticular nucleus, putamen; ROL, rolandic operculum; SFGdor, superior frontal gyrus, dorsolateral; SFGmed, superior frontal gyrus, medial; SOG, superior occipital gyrus; STG, superior temporal gyrus; TPOmid, temporal pole: middle temporal gyrus; TPOsup, temporal pole: superior temporal gyrus; VER4 and 5, Vermis_4_5*.

### Alterations of RSNs

**Figure 3:**
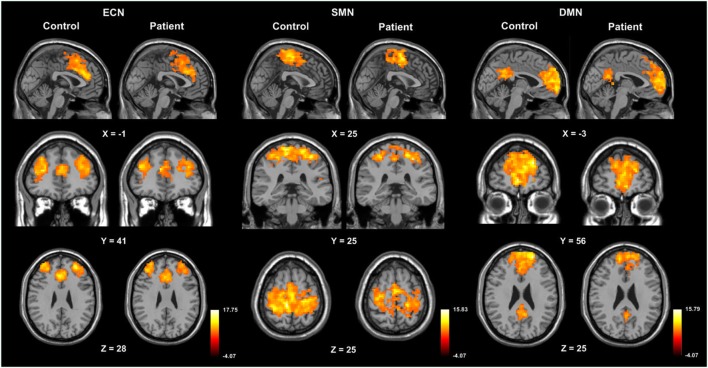
One-sample *t*-maps, illustrating activation of the default mode network (DMN), executive control network (ECN), and sensorimotor network (SMN) in control and patient groups.

The three RSN components (DMN, ECN, and SMN) anatomically neighboring the anterior CC exhibited greater inactivation in patients compared to healthy controls (Figure 3; Table 4).

**Table 4:** Significant differences of RSN between patients and controls.

Resting-state network	Regions	MNI coordinate	Peak *t*-score	Number of voxels
ECN	R superior frontal gyrus	18, 60, 21	−3.661	73
SMN	L postcentral gyrus	−27, −33, 72	−3.5747	120
	R postcentral gyrus	60, 0, 21	−3.4248	54
DMN	R superior frontal gyrus	21, 69, 9	−3.39	77
	L superior frontal gyrus	−18, 63, 18	−5.51	184
	Posterior Cingulate	−3, −33, 21	−4.44	131
	L precuneus	−6, −66, 33	−4.29	87

*The values given are the stereotactic MNI coordinates and the *t*-score of each anatomical region. SPM maps were thresholded at *p* < 0.05 (AlphaSim correction, voxel-level, minimum cluster size threshold of 54 voxels)*.

*ECN, executive control network; SMN, sensorimotor network; DMN, default mode network; R, right; L, left; MNI, Montreal Neurological Institute*.

## Discussion

The present study demonstrated functional connectivity alterations of local and remote networks in patients with epilepsy during the interictal period. The most notable finding of our network study was a significant increase in regional connectivity in the CC. Functional networks including the ECN, SMN, and DMN, all of which are anatomically associated with the CC, were inactive in the patient group.

The basic mechanism of seizure generalization involves intrahemispheric thalamocortical circuitry and interhemispheric CC. Tukel and Jasper were the first to describe the secondary synchrony in parasagittal lesions (18). The casollal and other interhemispheric connections are presumed to transmit unilateral ictal activities to the contralateral hemisphere. Wieshmann et al. performed MRI lesion mapping to investigate the relationship between the spatial extension of the oligodendrogliomas and seizure generalization. Their results suggested the genu of the CC as the major pathway for seizure generalization (19). Anatomically, this implication was consistent with our findings.

Structural CC pathologies comprising developmental abnormalities or microstructural changes in epilepsies have been reported. In addition to the visible lesion on brain images, DTI demonstrated microstructural CC changes in patients with IGEs and mTLE. A voxel-based DTI study in juvenile myoclonic epilepsy (JME) confirmed the reduced anisotropy in the CC. Focke et al. studied 25 patients with IGE (including 12 patients with subsyndrome JME) using TBSS and identified microstructural alterations in several large WM tracts, including the CC (4). Caligiuri et al. investigated the CC integrity in patients with benign TLE. They found significantly reduced thickness and FA of the anterior CC in right mTLE (20). Whelan and his colleagues used TBSS to identify significant FA reduction in the anterior CC from 25 patients with sporadic MRI-negative mTLE (21). Even though the patients in our study exhibited benign, MRI-negative lesions and had no DTI changes, we still found a functional change: increased regional synchronization in the anterior CC.

Temporal coupling or synchronization of neural activity among cerebral regions has been proposed as a critical mechanism for information integration that is mediated unilaterally by vast networks of intrahemispheric connections and between bilateral hemispheres by commissure fibers, especially the CC (22). Essentially, the intrahemispheric cortical synchronization may depend on the CC. Through bilateral hemispheric electrocortical recordings, Rojas-Ramos et al. demonstrated that the strength of the intrahemispheric temporal coupling changed significantly after callosal transaction. They proposed that callosal connections also play a role in local synchronization within unilateral hemispheres for abundant ipsilateral connections of callosal targets (3). In our patients, the regional synchronization of neural activity at the interictal resting state was changed over different cortical regions beyond the CC. In addition, significantly different inter- and intrahemispheric distant functional connectivity was observed in the correlation matrix analysis. However, the alterations of regional or distant synchronization had no associated cerebral morphormetric changes.

Of all prior ReHO studies of various epilepsy syndromes, only Zeng and colleagues found increased ReHo in the CC in epileptic patients with hippocampal sclerosis (7). In the other ReHo studies, no change of CC ReHo was found in patients with IGEs (8, 23–26). In addition, Zeng also demonstrated the reduction of ReHo in the DMN. They proposed that the increased ReHo in specific regions (e.g., hippocampus, midbrain, insula, and frontoparietal subcortical structure) may be related to a network that may be responsible for seizure genesis and propagation. Even though they did not study structural changes, similar to our findings, they reported that the regional hypersynchronization of the CC is an independent and default factor for seizure generalization.

Addressing the functional networks, the studied three RSNs were less active in patients than healthy controls. It is widely accepted that exogenous or endogenous stimulation of the brain will interrupt the resting state and cause the deactivation and suspension of the DMN (27–29). Based on the above theory, the interictal activity could be considered as internal stimulation, which may decrease or interrupt the DMN. Deactivation or suspension of the network activity in epilepsy has been reported by previous studies (30–32). This is proposed to be responsible for wide functional impairments in cognitive processes. The SMN is classified as one of the network groups involved in motor and sensory processes. There was a region over the right precentral gyrus overlapping with the SMN that exhibited significantly decreased ReHo in our patients. This finding may be consistent with the hypothesis based on previous studies for mTLE: decreased coherence in BOLD fluctuations in nodes of the networks contributed to impairment in functional connectivity (7).

There were limitations in this research. Without EEG monitoring during MR scanning, we could not measure the impact of interictal discharges on BOLD alterations. Strict selection of MRI-negative patients limited the sample numbers. In addition, low gradient directions might underestimate microstructural changes of CC. However, we observed the presence of a spatial specificity in the increased ReHo of CC. In epilepsy, the alteration of synchrony may be an antecedent to structural changes and correlate with the generalization of seizure in patients with focal epilepsies.

## Ethics Statement

The study protocol, which consisted of retrospectively analyzing the images of the patients and collecting images from the control subjects, was approved by the ethics committee of Buddhist Tzu Chi General Hospital in Hualien, Taiwan, and written informed consent was obtained from each participant.

## Author Contributions

Conceived and designed the study and wrote and revised the paper: S-JP and Y-LH. Performed the experiments: Y-LH. Analyzed the data: S-JP.

## Conflict of Interest Statement

The authors declare that the research was conducted in the absence of any commercial or financial relationships that could be construed as a potential conflict of interest.

## Footnotes

^1^http://www.fmrib.ox.ac.uk/fsl.

^2^http://surfer.nmr.mgh.harvard.edu.

^3^http://icatb.sourceforge.net/.
